# Analysis of adverse event reports associated with complementary health products in Singapore from 2017 to 2023

**DOI:** 10.3389/fmed.2026.1872698

**Published:** 2026-07-10

**Authors:** Yi-Ling Quek, Yun Zeng, Xiaowei Ge, Min-Yong Low, Suet-Leng P. Ng, Mun-Yee Tham, Sally Soh

**Affiliations:** 1 Pharmaceutical Division, Applied Sciences Group, Health Sciences Authority, Singapore, Singapore; 2 Vigilance and Compliance Branch, Health Products Regulation Group, Health Sciences Authority, Singapore, Singapore

**Keywords:** adulterants, adverse events, complementary health products, pharmacovigilance, skin and appendages disorders

## Abstract

**Introduction:**

The use of complementary health products (CHPs), including Chinese Proprietary Medicines (CPMs), health supplements, traditional medicines and homeopathic medicines, has become increasingly prevalent in Singapore's multicultural society. While these products are widely used for maintaining health and treating minor ailments and are generally safe, potential adverse effects have been reported. This study analyzes adverse event reports related to CHPs submitted to Singapore Health Sciences Authority (HSA) from 2017 to 2023.

**Methods:**

AE reports involving CHPs assessed by the Vigilance and Compliance Branch (VCB) of HSA from 2017 to 2023 were collated and analyzed. The analysis included patient demographics, AE details, suspected product information, medical history, laboratory results, concomitant therapies, and reporter's profession.

**Results:**

Of the 182,131 AE reports associated with pharmaceutical products and CHPs, 727 reports (0.4%) were associated with CHPs. Health supplements accounted for the highest number of reports (68% of total). “Skin and appendages disorders” were the most commonly reported system organ class, with glucosamine-containing products accounting for the highest number of adverse events. Patients primarily used CHPs for pain relief (33.1%), general health and wellbeing (20.6%), and weight management (8.8%). Products found to contain undeclared illegal substances were most frequently indicated for pain relief. The three most common adulterants were dexamethasone, chlorpheniramine, and prednisolone.

**Discussion:**

While CHPs are generally safe, adulterated products, especially those from dubious sources, pose real health risks. Healthcare professionals and consumers should remain vigilant about potential adulteration and report suspected CHPs related AEs. Even when causality remains unclear, reporting supports timely regulatory actions when concerning patterns emerge.

**Conclusion:**

This study examined 727 adverse event reports associated with CHPs between 2017 and 2023, accounting for 0.4% of all reported adverse events. Health supplements were the most frequently reported category, with glucosamine-containing products accounting for the highest number of adverse events, predominantly affecting the skin. Products found to contain undeclared illegal substances were most frequently indicated for pain relief, with dexamethasone, chlorpheniramine, and prednisolone being the most common adulterants found. These findings highlight the need for continued vigilance in the monitoring of CHPs to safeguard public health.

## 1 Introduction

The use of complementary health products (CHPs), including Chinese Proprietary Medicines (CPMs), health supplements, traditional medicines and homeopathic medicines, has become increasingly prevalent in Singapore's multicultural society (1, 2). These products are commonly used for maintaining general health and wellbeing, as well as treating minor ailments and symptoms. Singapore's unique position as a confluence of Eastern and Western medical practices is reflected in the widespread use of CHPs, with many residents incorporating these products into their healthcare regimens (1–3). Although these products are often perceived as inherently safe due to their natural origins and easy availability as over-the-counter medications, they can trigger adverse events ranging from mild discomfort to severe medical complications (4, 5). This risk is compounded when these products contain undeclared pharmaceutical substances or adulterants, which may cause serious harm without the consumer's knowledge. Most of the adverse event reports we received for adulterated products exhibit the known adverse events of the undeclared pharmaceutical substances or adulterants. Recognizing the growing global significance of herbal medicines, the World Health Organization (WHO) has established guidelines for safety monitoring within existing pharmacovigilance frameworks (6).

The Health Sciences Authority (HSA) oversees Singapore's pharmacovigilance system that monitors and evaluates safety concerns related to health products, including CHPs. The regulatory framework for CHPs in Singapore has progressively evolved, with the implementation of various measures such as mandatory product listing for CPMs, good manufacturing practice requirements and post-market surveillance to monitor the safety of CHPs (7–10). As part of post-market surveillance, understanding the patterns and characteristics of CHP-related adverse events is vital for public health safety and informed regulatory decision-making.

Several factors compound the complexity of CHP-related adverse events, including diverse product sources, varying manufacturing quality standards, potential contamination or adulteration with undeclared ingredients or harmful substances, and interactions with conventional medications or other herbal products (11–13). Moreover, cultural beliefs and practices surrounding CHPs use can influence reporting patterns and healthcare-seeking behavior among affected individuals (1, 3, 14).

Previous Singapore-based studies have employed data-mining techniques to evaluate CHP-related adverse events reported between 1998 and 2016 (15, 16). No updated analysis has been conducted since the 7-year period from 2010 to 2016. This study provides an updated pharmacovigilance analysis of CHPs in Singapore for the subsequent 7-year period from 2017 to 2023. The analysis aims to examine the trends in adverse reactions, product categories of concern, demographic patterns of affected individuals, and quality issues including adulterants found in the implicated products.

This study's findings will contribute to evidence-based recommendations for enhanced product safety monitoring, improved public awareness, and strengthened regulatory framework for CHPs in Singapore. Furthermore, this research will assist healthcare professionals in better understanding and managing CHP-related adverse events while guiding policy decisions to safeguard public health.

## 2 Methods

### 2.1 Source of data

This study is based on spontaneous adverse event reports associated with CHPs submitted to the HSA's Vigilance and Compliance Branch (VCB) in Singapore from 2017 to 2023. Adverse event reports could be submitted through multiple channels such as online submission, e-form, electronically *via* the Critical Medical Information Store (CMIS) module of the electronic medical record system, or by completing the “Suspected Adverse Events” form sent through postal mail or e-mail.

**Figure 1:**
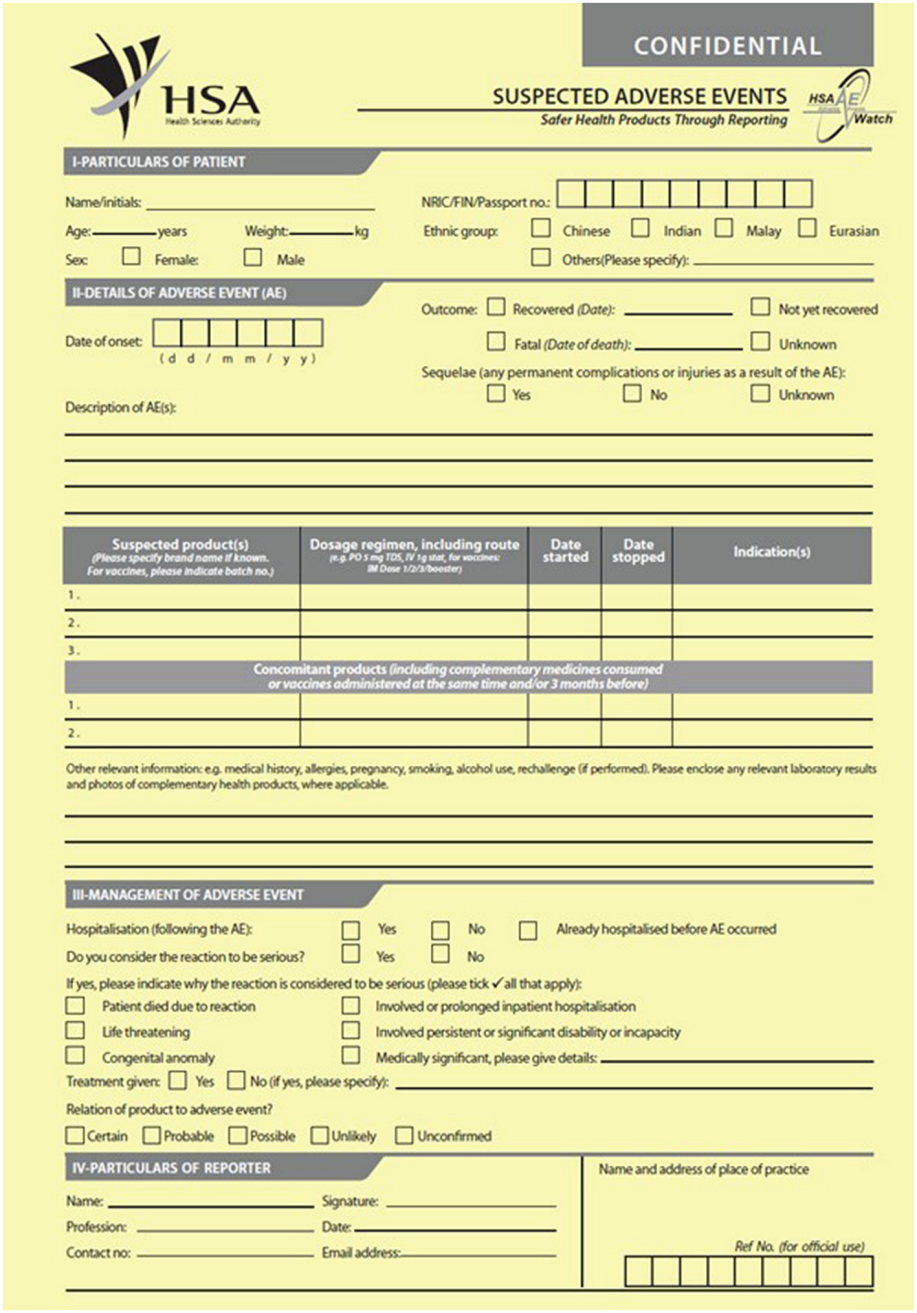
Suspected adverse events form.

The form, shown in Figure 1 (17), captures comprehensive information including patient details (name, gender, age, ethnicity, and identification number), a concise description of the adverse event, date of onset, seriousness, and patient outcome. It also documents information about the suspected health product (active ingredient, brand name, batch number, dose given, route of administration, therapy dates, and indications) and the reporter's details (name, contact number, and place of practice). Reporters, who may be healthcare professionals or companies, can indicate the causality of the adverse event as “certain”, “probable”, “possible”, “unlikely”, or “unconfirmed”.

Upon receiving a report, VCB regulatory specialists would evaluate the information, and follow up with the reporters for any missing or unclear information. Any duplicate reports identified would be removed. The case is then reviewed by at least 2 VCB regulatory specialists to determine the seriousness of the adverse event and to assess causality between the product and the adverse event, with reference to WHO-Uppsala Monitoring Center (UMC) causality assessment criteria (18). These reports are then stored in the Pharmaceutical Regulatory Information System (PRISM) database for periodic aggregation and analysis to detect potential safety signals. In cases where the product is suspected to contain adulterants or heavy metals, testing of the sample could also be considered. When necessary, HSA would implement appropriate regulatory actions, such as communications to healthcare professionals or the public, product recalls or suspension or cancellation of product registration.

Healthcare professionals and industry partners could access the PRISM for information about suspected local adverse events involving therapeutic products and CHPs. As a full member of the WHO Programme for International Drug Monitoring, these adverse event reports were also submitted to the Uppsala Monitoring Center in Sweden for inclusion in WHO's VigiBase (19).

### 2.2 Data collation and analysis

The study examined adverse events associated with CHPs, specifically CPMs, health supplements and other complementary medicines (including Malay traditional medicine, Jamu, Ayurvedic medicine, and homeopathic medicine) from January 2017 to December 2023, with data generated on 13 March 2024. In this study, CPM is defined as a medicinal product that contains herbs, animal parts or minerals documented for use in the practice of TCM and is in the form of a finished product, such as a capsule or tablet (8). Health supplement is defined as a product that is used to supplement a diet and to support healthy functions of the human body and contains one or more of the following ingredients: vitamins, minerals, amino acids and substances derived from natural and botanical sources, and presented in dosage forms to be administered in small unit doses such as capsules, softgels, tablets, powders, and liquids (7). Other types of complementary medicines refer to traditional medicines that contain ingredients with uses documented in the relevant Traditional Indian/Malay references and presented in dosage forms to be administered in small unit doses such as capsules, softgels, tablets, powders, and liquids (9).

During this period, HSA received a total of 182,131 adverse event reports associated with pharmaceutical products and CHPs, of which 727 reports were related to CHPs. All CHP-related adverse event reports meeting the inclusion criteria during the study period from 2017 to 2023 were included in the analysis. The inclusion criteria included reports approved and assessed by VCB with a WHO-UMC causality assessment of “certain”, “probable”, or “possible”. Reports outside the study period, reports unrelated to CHPs, and reports with a causality assessment of “unlikely” or “unconfirmed” were excluded. The following information was extracted from the reports and collated in Microsoft^®^ Excel for analysis: age, gender, ethnicity of patient, type and indication of product, route of administration, organ system affected, hospitalization status, seriousness and outcome of adverse event, medical history, concomitant medications, laboratory results, and profession of the reporter. As spontaneous adverse event reports are submitted on a voluntary basis, not all variables were available for all reports. No patient identifiers were included in the extracted information.

Adverse events were categorized according to the WHO's adverse reaction terminology (WHO-ART) into various system organ classes (SOCs) (20). Multiple product types, adverse events or system organ classes could be described in a single adverse event report. Patient hospitalization status was classified as “hospitalized”, “not hospitalized”, or “already hospitalized”. The term “already hospitalized” referred to patients admitted for other conditions prior to the adverse event. Outcomes of adverse events were grouped into four categories as “recovered”, “not yet recovered”, “uncertain outcome”, and “died”. Any other medicines taken within 3 months of reporting were considered concomitant medications.

To investigate possible products suspected of adulteration, implicated products with a high index of suspicion for the presence of adulterants or heavy toxic metals were sent to the HSA's Pharmaceutical Laboratory to screen for these substances. Screening of adulterants was conducted using gas chromatography/mass spectrometry (GC/MS) and high performance liquid chromatography/diode array detection (HPLC/DAD) (21). Test results were collated, and information on the adulterant types and frequency was compiled for adulterated products. Descriptive statistics were used to analyze the variables collected from the reports.

This study utilized de-identified data collected through routine public health safety surveillance activities. As such, it does not constitute human subjects research and was therefore exempt from institutional review board review.

## 3 Results

### 3.1 Characteristics of adverse event reports

During the period from 2017 to 2023, HSA received a total of 182,131 adverse event reports associated with pharmaceutical products and CHPs, of which 727 reports were associated with CHPs, consisting of CPMs, health supplements and other types of complementary medicines. It should be noted that this figure reflects the number of reports submitted to HSA and not the actual number of adverse events occurring in the community. Each adverse event report could include more than one product type or suspected drug, more than one adverse event and more than one system organ class.

**Figure 2:**
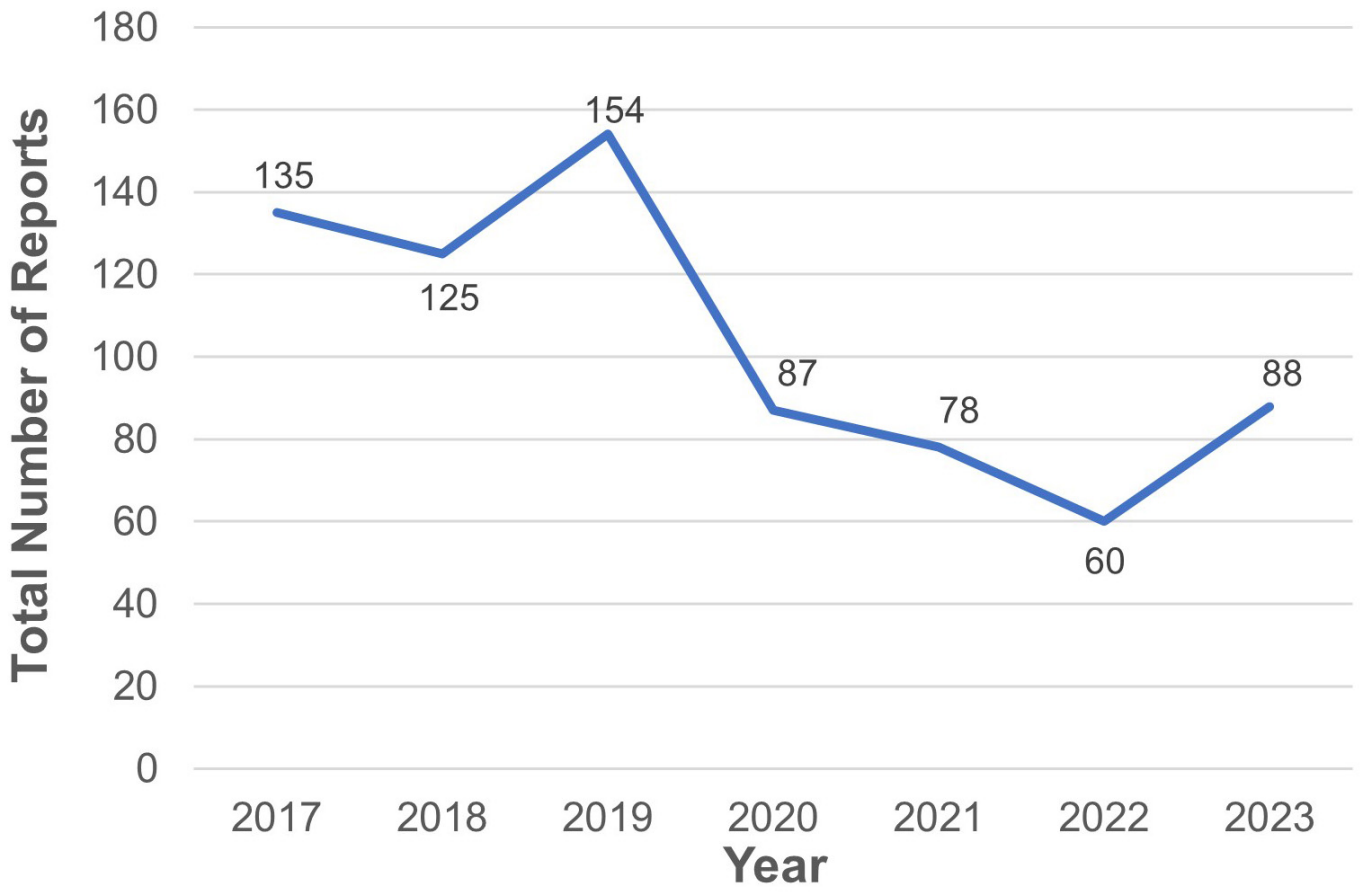
Total number of adverse event reports associated with complementary health products for the period 2017–2023.

Table 1 presents the annual distribution of adverse event reports from 2017 to 2023, comparing the total number of adverse event reports with the number of reports associated with CHPs. CHP-related reports accounted for 727 out of 182,131 reports, representing approximately 0.4% of all adverse event reports received during the study period. Figure 2 shows that the highest number of CHP-related reports occurred in 2019, with 154 reports, while the lowest number occurred in 2022, with 60 reports. In 2023, the number increased again to 88 reports.

**Table 1:** Number of adverse event reports associated with different types of complementary health products for the period 2017–2023.

Year	Total no. of adverse event reports associated with pharmaceutical products and complementary health products	Total no. of adverse event reports associated with complementary health products	Number of adverse event reports associated with different types of complementary health products^*^		
			Chinese proprietary medicines	Health supplements	Other types of complementary medicines
2017	21,022	135	22	86	29
2018	23,906	125	13	75	41
2019	36,187	154	7	123	26
2020	20,465	87	10	59	21
2021	34,516	78	6	55	17
2022	20,572	60	7	43	10
2023	25,463	88	13	54	22
Total	182,131	727	78	495	166

^*^There may be more than 1 product type, adverse event or system organ class involved in each adverse event report. Hence, the sum of reports across these product types will exceed the total number of reports.

Of the 727 CHP-associated cases, health supplements accounted for the highest number of reports (68.1%, *n* = 495), while CPMs comprised 10.7% (*n* = 78) of the reports. Malay traditional medicine, Jamu, Ayurvedic medicine, and homeopathic medicine collectively made up the remaining 22.8% (*n* = 166) of the reports. There were 297 (41%) reports classified as serious by the VCB, including three fatalities. Among the 297 serious adverse event reports, health supplements accounted for the highest proportion at 46.4%, followed by Malay traditional medicine, Jamu, Ayurvedic medicine, and homeopathic medicine, which collectively comprised 33.8%, and CPMs at 19.8%. 45 complementary medicine cases (27.1%; of which 16 cases were related to Malay traditional medicines) and eight CPM cases (10.3%) were found to contain undeclared drugs. In contrast, only three health supplement cases (0.6%) showed evidence of adulteration.

**Figure 3:**
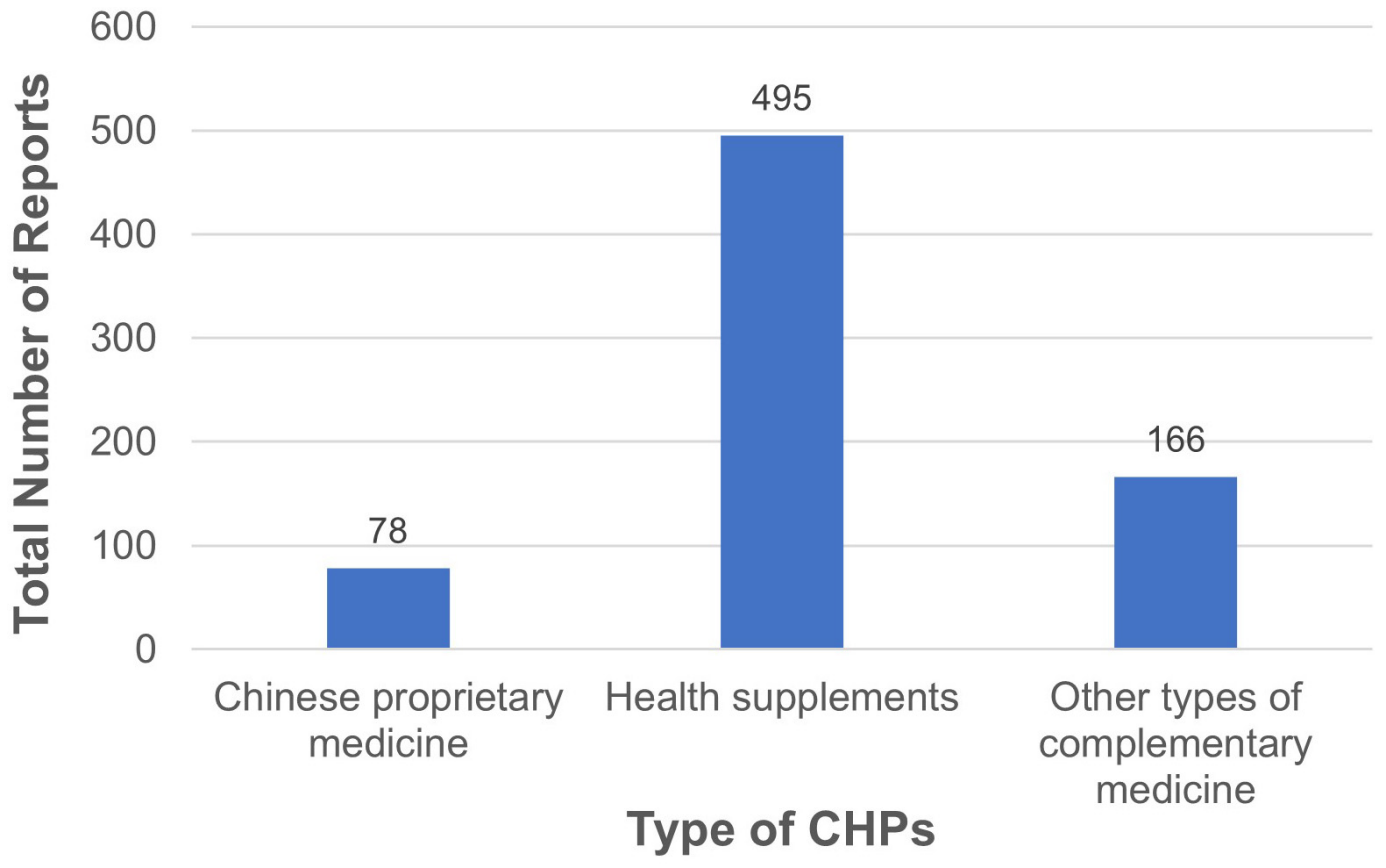
Total number of adverse event reports associated with different types of complementary health products for the period 2017–2023.

Based on Figure 3, health supplements were the product category most frequently reported, accounting for 495 reports. This was followed by other types of complementary medicines with 166 reports, and Chinese proprietary medicines with 78 reports. This result indicates that between 2017 and 2023, adverse event reports related to CHPs in Singapore were most commonly reported with health supplements.

Among the 495 health supplement-related reports, 360 (72.7%) involved glucosamine-containing products, describing hypersensitivity reactions such as rash, pruritus, angioedema, urticaria, and periorbital and facial oedema.

### 3.2 Patient demographics, profession of reporter and source of reporting

Table 2 presents patient demographics (gender, age group, and ethnicity), profession of reporter and source of reporting. Not all reports contained complete information. Where reported, female patients accounted for 459 (63.1%) cases, while male patients comprised 243 (33.5%) cases, demonstrating a higher reporting rate for women. The highest incidence of adverse events was observed for elderly adults above 60 years old (40.9%), with older adults aged 41–60 years old representing the second-largest group (30.5%). In terms of ethnicity, Chinese patients constituted the majority (59.6%), followed by cases of unknown ethnicity (16.6%) and Malay patients (15.5%). Indian patients and those of other ethnicities represented smaller proportions at 5.1 and 3.2% respectively. Adverse event reports associated with health supplements were the highest among all three ethnic groups, following similar trends: Indians (73.0%), Chinese (63.5%) and Malays (62.8%). Adverse events linked to other types of complementary medicines were the second most commonly reported across the ethnicities: Malays (33.6%), Indians (27.0%) and Chinese (20.8%). Adverse events associated with CPMs were predominantly reported by the Chinese (15.7%), with minimal usage among Malays (3.5%) and no usage among Indians. Healthcare professionals submitting these reports were predominantly doctors (89.9%) and pharmacists (8.9%). Public hospitals and polyclinics were the main source of adverse event reports (86.9%), followed by private hospitals/clinics (12.1%), pharmaceutical companies (0.7%), and retail pharmacies and others (0.3%).

**Table 2:** Patient demographics, profession of reporter and source of reporting of a total of 727 adverse event reports.

Patient demographics	No. of reports, *n* (%)	Reporting source	No. of reports, *n* (%)
Gender		Profession of reporter	
Female	459 (63.1)	Doctor	654 (89.9)
Male	243 (33.5)	Pharmacist	65 (8.9)
Information not available	25 (3.4)	Manufacturer	4 (0.6)
		Nurse	4 (0.6)
Age		Source of reporting	
<1	11 (1.5)		
1–20	44 (6.1)	Public hospitals/polyclinics	632 (86.9)
21–40	81 (11.1)	Private hospitals/clinics	88 (12.1)
41–60	222 (30.5)	Pharmaceutical companies	5 (0.7)
>60	297 (40.9)	Retail pharmacies, others	2 (0.3)
Information not available	72 (9.9)		
Ethnicity			
Chinese	433 (59.6)		
Malay	113 (15.5)		
Indian	37 (5.1)		
Others	23 (3.2)		
Information not available	121 (16.6)		

### 3.3 Indications, route of administration and concomitant medication

Of the 727 cases studied, only 160 (22%) documented the indications for CHP usage. Within these 160 cases, the majority of patients used CHPs for pain relief (53 cases, 33.1%), for conditions such as gout, joint pain, neck pain, shoulder discomfort, hand pain, knee problems, back pain, and headaches. Other reported indications included general health and wellbeing (33 cases, 20.6%), weight management (14 cases, 8.8%), skin conditions including itch, eczema and rashes (8 cases, 5%), cough (7 cases, 4.4%), diabetes (5 cases, 3.1%), hypertension (4 cases, 2.5%), liver health (3 cases, 1.9%), and sexual performance enhancement (2 cases, 1.3%).

Regarding administration methods, 235 patients (32.4%) took the products orally in various forms including tablets, capsules, powder, or liquid. Six cases (0.8%) applied products topically, and one case (0.1%) administered the product through inhalation. The route of administration was not recorded for the remaining 485 cases (66.7%). Only 57 patients (7.8%) reported taking both CHPs and conventional medications concurrently during the study period, while no concomitant medication use was reported in the remaining 670 cases (92.2%).

### 3.4 Hospitalization, outcomes of adverse events and causality assessment

Of the 727 adverse event reports, 99 patients (13.6%) required hospitalization due to adverse events, 13 patients (1.8%) were already hospitalized at the time of reporting, and 65 patients (8.9%) were treated as outpatients. The hospitalization status of the remaining 550 patients (75.7%) was not documented. Regarding outcomes of adverse events associated with CHP usage, 98 patients (13.5%) recovered at the time of reporting, while 82 (11.3%) were still recovering. The outcome remained uncertain for 544 patients (74.8%), and three patients (0.4%) died. Of the fatalities, one patient had developed liver injury with raw herbs, one had drug reaction with eosinophilia and systemic symptoms (DRESS) with a Malay traditional medicine obtained from Malaysia and another had brain hemorrhage after consuming eight types of slimming products a friend had purchased from Thailand. The case of liver injury may be attributed to the possible inherent hepatotoxic properties of the herbs. For the remaining two cases, adulteration could not be established as the samples were not available for testing. However, for the Malay traditional medicine obtained from Malaysia, Malaysia's National Pharmaceutical Regulatory Agency (NPRA) listed a product with a similar name as being adulterated with “chlorpheniramine and dexamethasone”.

Of the 727 adverse event reports analyzed, causality assessments were categorized as “certain” in 28 reports, “probable” in 47 reports, and “possible” in 652 reports. It should be noted that these causality assessment categories reflect the degree of likelihood of an association between the CHP and the reported adverse event, rather than definitive proof of causation. The predominance of reports classified as “possible” is consistent with the inherent limitations of spontaneous adverse event reporting, where incomplete clinical information, the presence of concomitant medications, and underlying comorbidities may make it difficult to establish a stronger causal link.

### 3.5 Adverse events grouped according to system organ class classification

Table 3 presents the system organ class (SOC) classification of adverse events associated with CHPs. One report may include more than one adverse event or more than one system organ class, so the total number of adverse events may exceed the total number of CHP-related reports. The 727 reports documented 859 distinct adverse events; percentages were calculated based on the number of reports. The predominant SOC was “skin and appendages disorders” (432 reports, 59.4%), with 417 (57.4%) cases involving hypersensitivity reactions such as rash, pruritus, angioedema, urticarial, and periorbital oedema. Glucosamine-containing products made up most of the cases (65%), followed by melatonin-containing products (6.7%) and products containing dried ivy leaf extract (6.3%).

**Table 3:** System Organ Class (SOC) classification of adverse events associated with complementary health products arranged in decreasing order of prevalence.

No.	SOC involved	No. of adverse event reports, n^*^
1.	Skin and appendages disorders	432
2.	Body as a whole—general disorders	91
3.	Liver and biliary system disorders	57
4.	Endocrine disorders	42
5.	Urinary system disorders	40
6.	Gastro-intestinal system disorders	36
7.	Metabolic and nutritional disorders	28
8.	Respiratory system disorders	26
9.	Central and peripheral nervous system disorders	23
10.	Heart rate and rhythm disorders	21
11.	Musculo-skeletal system disorders	16
12.	Cardiovascular disorders, general	12
13.	Vascular (extracardiac) disorders	8
14.	Psychiatric disorders	7
15.	Vision disorders	6
16.	Platelet, bleeding and clotting disorders	4
17.	Myo-, endo-, and peripheral and valve disorders	3
18.	Resistance mechanism disorders	2
19.	White cells and reticuloendothelial system disorders	1
20.	Reproductive disorders, female	1
21.	Others	3
	Total	859

^*^Number of adverse event reports in each system organ class. One report may include more than one adverse event or more than one system organ class.

The second most frequent SOC was “body as a whole – general disorders” (91 reports), with symptoms like pain, oedema, fever, anaphylactic reaction, and malaise. Ten reports were anaphylactic reactions, including four reports attributed to probiotics. Ten reports were linked to CPM products and complementary medicines adulterated with undeclared drugs, including dexamethasone and chlorpheniramine.

The third most reported SOC was “liver and biliary system disorders” (57 reports). A significant case involved a complementary medicine called “PILL HUA LUO CIN TAN”, which was found to contain undeclared potent steroids (dexamethasone and prednisolone) and a painkiller (diclofenac) (22). A man in his 50s experienced hepatitis relapse, with a sudden increase in liver inflammation after consuming this product for approximately 1 month. He also developed Cushing's syndrome symptoms including facial puffiness and abnormal blood cortisol levels, leading to hospitalization. The patient had purchased the product from a “traditional Chinese medicine shop” in Malaysia for wrist pain.

Another herb Chai Hu (*Radix Bupleuri*) was identified in 7 of the 57 adverse event reports associated with “Liver and biliary system disorders”. These cases, involving Chinese proprietary medicines containing *Radix Bupleuri*, resulted in liver injury (2 reports), hepatitis (2 reports), abnormal hepatic function (2 reports), and jaundice (1 report), suggesting the herb's potential hepatoxicity (23).

The fourth most reported SOC was “endocrine disorders” (42 reports), largely driven by Cushing's syndrome associated with CHPs adulterated with steroids.

Serious adverse events during the study period included Cushing's syndrome (32 reports), liver injury (15 reports), anaphylaxis (10 reports), acute renal failure (6 reports), hypoglycemia (3 reports), Stevens-Johnson syndrome (2 reports), DRESS syndrome (1 report), brain hemorrhage (1 report), exfoliative dermatitis (1 report), hepatic failure (1 report) and toxic epidermal necrolysis (1 report). Among the 32 patients with Cushing's syndrome, 26 had consumed CHP products adulterated with various drugs, including steroids (dexamethasone, prednisolone, betamethasone, and clobetasol propionate), antihistamines (chlorpheniramine, promethazine, and cetirizine), diuretics (frusemide), non-steroidal anti-inflammatory drugs (piroxicam, indomethacin), antifungals (griseofulvin), and painkiller (diclofenac, paracetamol). These products were primarily used for pain relief related to gout, joint, knee, foot and back, and for skin conditions such as eczema or rashes.

### 3.6 Adulterants

During 2017–2023, laboratory testing was conducted on 73 (10%) products out of 727 CHPs. The remaining products were not tested due to various factors, including product unavailability, insufficient evidence linking adverse events to the products, and products which are already known to contain adulterants based on publicly available information. Among the tested products, 56 (76.7%) products were found to contain undeclared drugs, while no drug adulterants were detected in the other products. The adulterated products comprised three health supplements, eight CPMs and 45 other types of complementary medicines. As shown in Table 4, the most frequently detected adulterants were dexamethasone, chlorpheniramine, and prednisolone, in descending order. Of the 56 adulterated products, 36 products contained pain-relief drugs such as diclofenac, paracetamol, piroxicam, and ibuprofen, as well as oral steroids such as dexamethasone and prednisolone. Eight products contained slimming drugs such as sibutramine. Five products contained steroids for treating eczema and rashes, of which three products contained topical steroids such as betamethasone and clobetasol propionate, while two products contained oral steroids such as dexamethasone and prednisolone. Four products contained erectile dysfunction drugs including sildenafil and tadalafil.

**Table 4:** Adulterants (in alphabetical order) detected in complementary health products, with the frequencies of the three most commonly detected drug adulterants in bold.

Adulterants	Frequency^*^
Amoxycillin	1
Atorvastatin	1
Betamethasone	2
Cetirizine	1
Chloramphenicol	3
Chlorpheniramine	**18**
Clobetasol Propionate	1
Clotrimazole	1
Cyproheptadine	1
Dexamethasone	**40**
Diclofenac	7
Diphenhydramine	1
Furosemide	6
Griseofulvin	7
Ibuprofen	3
Indomethacin	1
Ketoconazole	1
Lovastatin	2
Paracetamol	8
Piroxicam	9
Prednisolone	**10**
Promethazine	2
Salicylic acid	1
Sibutramine	8
Sildenafil	3
Tadalafil	1
Tetracycline	3

^*^A product could be adulterated with more than 1 adulterant.

Table 5 indicates that 38 out of the 56 adulterated products contained multiple adulterants. Twelve products had two adulterants each, 11 products had 3 adulterants each, another 11 products had 4 adulterants each, one product had five adulterants, and three products had six adulterants each. One notable example of a product containing six adulterants was a CPM called “Traditional Herbs Preparation XPE”. This product contained dexamethasone (a steroid), chlorpheniramine (an antihistamine), ibuprofen (a non-steroidal anti-inflammatory drug), lovastatin (a cholesterol-lowering medicine), chloramphenicol, and tetracycline (both antibiotics) (24). A Chinese woman in her 60s who consumed this product for over 9 months for joint pain reported worsening symptoms when attempting to reduce or cease usage.

**Table 5:** Frequency of complementary health products with different number of undeclared drug adulterants detected.

No. of adulterants detected	No. of products (*n* = 56)
1	18
2	12
3	11
4	11
5	1
6	3

Another significant case involved “PHQ 1001 Khasiat Penawar Herba Qaseh Serata Herb”, a complementary medicine found to contain four adulterants, namely dexamethasone, piroxicam, griseofulvin, and paracetamol (25). A Malay woman in her 60s who took this product for knee pain developed complications including rapid weight gain and facial puffiness after several months of use. She was subsequently diagnosed with diabetes and Cushing's syndrome, likely caused by the dexamethasone present in the product. Similar weight gain was reported by several of her friends who also used the product, presumably due to its steroid content.

In another report, a woman in her 40s used “Lung Tan Tsao”, a CPM, for eczema treatment but developed weight gain and facial swelling. Subsequent testing revealed the presence of chlorpheniramine and dexamethasone in the product (26). In another case, a woman in her 40s developed an intractable cough and abnormal blood cortisol levels after 2 months of using a CPM “Yanwo Chongcao Yanyin Qinfei Huatan Dan” (27). Analysis showed that this product contained dexamethasone, prednisolone, chlorpheniramine, and promethazine.

Another woman in her 60s developed Cushing's syndrome after consuming “CHONG CAO DAN” for 2–3 years (28). The product, taken for muscle pain relief, was found to be adulterated with chlorpheniramine, dexamethasone, and furosemide, resulting in round face, skin thinning, and worsened hypertension. In another case, a man in his 60s developed Cushing's syndrome with round face and weight gain, after taking a CPM “AlphaMiracHERBS” for psoriasis over 3–4 months (29). Despite claims of being a “100% vegetarian” herbal supplement, laboratory tests revealed that the product contained six drugs, namely dexamethasone, chloramphenicol, tetracycline, chlorpheniramine, ibuprofen, and lovastatin. Fortunately, the man's condition improved after discontinuing the product.

A woman in her 50s suffered steroid-induced Cushing's syndrome after consuming unlabelled capsules purchased from a Singapore market peddler, for 3–4 months to treat her headache (30). The product, advertised as “100% Herbal” with claimed benefits against cancer, hypertension, and diabetes, was found to contain dexamethasone, prednisolone, chlorpheniramine, diclofenac, amoxicillin, and sildenafil. Similarly, a man in his 30s developed Cushing's syndrome after taking a CPM product “DND Rx9” for gout and arthritis (31), obtained from Malaysia. Testing revealed the presence of potent steroids like dexamethasone and prednisolone, as well as diclofenac. It is worth noting that unsupervised use of diclofenac can cause gastric damage and bleeding.

A notable case involved a woman in her 40s who experienced tachycardia, breathlessness, and dizziness after consuming “Clinic K”, a complementary medicine marketed for weight loss (32). While the product claimed to contain only natural ingredients, such as amino acids, green tea extract, and other botanical extracts, laboratory analysis revealed sibutramine levels at twice the previously permitted maximum daily dose. An even more serious case occurred in 2019, when a woman in her 50s developed life-threatening complications after taking “BB Body”, an adulterated slimming product, for 3 months (33). She experienced ventricular tachycardia, leading to unconsciousness, and required emergency resuscitation. The patient underwent intubation and cardioversion and subsequently diagnosed with non-ischemic cardiomyopathy. Her treatment required defibrillator implantation and long-term heart failure medication.

### 3.7 Toxic heavy metals

In this study, one product was found to contain toxic heavy metals exceeding Singapore's legal permissible limits. The product, “EUZEMA Confidence Revival Cream”, sold on a Malaysia-based retail website and Carousell (an e-commerce platform in Singapore) for eczema relief, contained arsenic levels more than 430 times above the allowable limit (34). A man in his 30s developed purpura, a severe skin reaction, after using this product for his eczema over the course of a year. His doctor suspected the adverse reaction was caused by “realgar”, an arsenic-containing mineral listed in the cream's ingredients and subsequently reported this incident. Arsenic, being a toxic heavy metal, can cause skin irritations, contact dermatitis, and rashes that result in skin peeling when applied topically. Extended, widespread application of products containing high arsenic levels may lead to arsenic poisoning. Besides arsenic, the cream was also found to contain betamethasone, a potent steroid, and salicylic acid, an exfoliating agent.

## 4 Discussion

### 4.1 Characteristics of adverse event reports

This study analyses trends in reported adverse events associated with CHP usage in Singapore over a 7-year period from January 2017 to December 2023. Singapore consistently records approximately 20,000 adverse event reports annually, which has remained stable over the past 14 years from 2010 to 2023 (16). Out of the 182,131 adverse event reports received by HSA during 2017–2023, only 727 reports were related to CHPs. This study describes the pattern of adverse event reported with CHPs in Singapore, rather than the actual incidence of adverse events in the community.

The study results show that health supplements were the most frequently reported product type in the adverse event reports with CHPs, with 495 reports, followed by other types of complementary medicines with 166 reports and Chinese proprietary medicines with 78 reports. For health supplements which accounted for the highest number of reports, there was only a moderate decrease to 495 reports (68%) during the 7-year study period compared to 625 reports (70%) in the previous 7-year period (16). This sustained level of reporting is more likely to be reflective of the continued popularity of health supplements in Singapore with the corresponding reporting trend of adverse events.

The highest number of serious adverse drug reactions were associated with health supplements, representing nearly half (46.4%) of all serious events reported. Their widespread availability through pharmacies, health stores, and online platforms leads to higher consumption rates and consequently higher reporting rates of adverse events (35). Consumers may also perceive these products as inherently safe due to their marketing as “natural products”, leading to prolonged use, or concurrent use with multiple products without professional guidance (36).

Glucosamine-containing supplements accounted for 72.7% of adverse event reports, primarily due to hypersensitivity reactions (rash, angioedema, pruritus, and urticaria). This represented a slight decrease from 82.7% recorded between 2010 and 2016 (16). Several factors could have contributed to the high prevalence of glucosamine-related adverse events. Glucosamine supplements, widely available in Singapore's hospitals, clinics, retail pharmacies, and online platforms, have gained popularity as a potential treatment for joint pain and arthritis, being generally considered safe (37, 38). This accessibility may lead to increased usage without adequate consideration of potential risks. Moreover, as glucosamine is typically derived from shellfish, it may trigger allergic reactions in sensitive individuals (39). This manufacturing origin creates an inherent allergenic potential that may not be immediately apparent to consumers or adequately communicated through product labeling. These findings highlight the need for improved adverse event awareness regarding shellfish-derived supplement risks among consumers, given glucosamine's widespread availability and perceived safety profile.

In the absence of exposure data to calculate the incidence of adverse events with the different product categories, it remains to be determined whether health supplements are associated with the highest risk of adverse events.

Adverse event reports associated with CPMs showed a slight increase to 78 reports (10.7%) compared to 72 reports (8.1%) in the previous 7-year period (16). The ethnic breakdown of reports aligned with Singapore's demographics (40), with Chinese patients accounting for 87.2% of the reports, reflecting cultural ties to Traditional Chinese Medicine and the integration of CPMs into health and wellness routines within Singapore's Chinese community (1, 3).

### 4.2 Patient demographics

#### 4.2.1 Age

Patients aged above 60 years old constituted the largest proportion (40.9%) of adverse event reports, aligning with findings by Xu et al. and Zazzara et al. (16, 41). This demographic typically presents multiple chronic medical conditions requiring various medications, thereby increasing their susceptibility to adverse drug reactions due to polypharmacy (41, 42). Elderly patients are likely to self-medicate by taking CHPs for pain relief, often under the misconception that these products are inherently safer, while being unaware of potential side effects (43). The combination of CHPs with their existing medication regimen may lead to dangerous drug-herb interactions and subsequent adverse reactions (44).

Age-related decline in liver and kidney function among elderly patients would affect drug clearance, potentially increasing the likelihood of adverse reactions (41). These physiological changes influenced how medications were absorbed, distributed, metabolized, and eliminated from the body.

#### 4.2.2 Gender distribution

Females accounted for nearly twice the number of adverse events (63.1%) compared to males (33.5%) in this study. These findings aligned with other reports, including the previous 7-year period study and a recent Korean study on adverse events associated with herbal medicine products, where women reported more than twice as many adverse events as men (16, 45–48). Cultural factors and social norms likely contributed to gender reporting disparity. Research suggested that women typically demonstrate greater health awareness and are more inclined to report adverse events compared to men, who may be less proactive in seeking medical advice or documenting side effects (49).

Additional factors include hormonal differences, drug utilization patterns, and biological variations (48, 50). Biological distinctions, particularly hormonal fluctuations and metabolic differences, may also affect drug processing in the body, potentially leading to varying adverse reactions between gender.

The higher reporting rate among females could be attributed to their increased usage of CHPs relative to males (1, 45, 49, 51). Women typically consume more CHPs, often for wellness, weight management, or aesthetic purposes, potentially increasing their exposure to associated risks and side effects.

#### 4.2.3 Ethnicity

The ethnic distribution of patients in CHP-related adverse event cases closely mirrored Singapore's demographic composition. With Chinese residents comprising 74% of Singapore's population (40), it was unsurprising that they accounted for the largest proportion of adverse event reports at 59.6%. The proportion of adverse events reported among Malay and Indian populations at 15.5 and 5.1% respectively, similarly aligned with their respective population shares of 13.5 and 9% (40). This proportional distribution suggests relatively consistent reporting behaviors across ethnic communities, indicating that cultural factors do not significantly influence the likelihood of reporting adverse events.

### 4.3 System organ class (SOC) classification

According to Table 3, “skin and appendages disorders” was the most frequently reported SOC for adverse events associated with CHPs. The predominant clinical manifestation was rashes, which aligned with findings from Xu et al.'s previous study (16). Similarly, analysis of the WHO adverse drug reaction database, VigiBase, revealed that 19.9% of adverse event reports related to traditional medicines were associated with the SOC of skin and subcutaneous tissue disorders (52). In this study, most skin and hypersensitivity reactions were associated with glucosamine-containing products (281 reports).

This observation could be attributed to both product composition and route of administration involving direct skin exposure. These dermatological adverse events primarily stem from the inherent properties of CHP ingredients, particularly in patients with pre-existing allergies. Many CHPs, encompassing herbal remedies and topical preparations, contain active ingredients capable of inducing allergic reactions or irritations (53, 54). Plant-based extracts, despite being marketed for their natural properties, contain compounds known to trigger skin sensitivities, ranging from rashes and redness to hives, and contact dermatitis (53, 54). Moreover, CHPs frequently incorporate preservatives, colorants, and synthetic additives that may prove unsuitable for certain skin types, potentially resulting in irritant or allergic contact dermatitis. Repeated product use can compromise the skin's natural barrier, increasing susceptibility to adverse reactions (55).

The route of administration further compounds these risks. Topical preparations create direct prolonged contact between potentially sensitizing substances and cutaneous tissues, triggering localized skin reactions when they are applied. In addition, the skin serves as the primary contact point for CHP exposure, regardless of the intended administration route, whether oral or topical, making dermatological symptoms the most readily observable manifestations of adverse reactions to these products. These dermatological issues may be intensified by underlying conditions, genetic factors, or incorrect product usage, contributing to their prevalence among CHP-related adverse events (56).

The frequency distribution of SOCs for adverse events related to herbal medicine products reported in Korea and Taiwan differed slightly from those observed in Singapore (45, 46). While gastrointestinal system disorders were the most frequently reported SOC in Korea and Taiwan, skin and subcutaneous tissue disorders remained highly prevalent as the second most common category, demonstrating the consistent significance of dermatological reactions across these Asian populations.

“Body as a whole – general disorders” was the second most reported SOC. The symptoms are generally broad and non-specific, including pain, body aches, fatigue, oedema, anaphylactic reaction, malaise, and fever, which could be an allergic reaction, or the individual's intolerance to the drug. There were 10 anaphylactic reactions within this SOC, constituting approximately 11% of reports in this category. Four anaphylactic reactions were attributed to probiotic products. Given that cow's milk proteins are found in some probiotic products (i.e., labeled as casein, whey, lactoglobulin, lactoferrin, or colostrum), its use in individuals with cow's milk allergy may trigger allergic reactions, including anaphylaxis (57).

“Liver and biliary system disorders” ranked as the third most commonly reported SOC in adverse events related to CHPs, primarily due to the impact of herbal and dietary supplements on liver function. Many CHPs, especially those containing plant-based compounds, contained active ingredients that may be metabolized by the liver, potentially causing hepatotoxicity in some cases (58). While herbs such as kava, comfrey, and black cohosh, for example, have been historically linked to liver damage (58, 59), these hepatotoxic herbs were not implicated in the hepatotoxic adverse events in this study.

Analysis of 57 adverse event reports associated with “Liver and biliary system disorders” revealed that seven cases were associated with Chinese proprietary medicines containing Chai Hu (*Radix Bupleuri*). The reported liver complications included liver injury, hepatitis, abnormal hepatic function, and jaundice. Studies have demonstrated that the toxicity of *Radix Bupleuri* primarily affects the liver, especially with prolonged usage, although liver function typically returns to normal upon discontinuation (23, 60). The herb had been linked to numerous cases of acute hepatitis, both when used independently and as a component of the Xiao-Chai-Hu-Tang formulation (known as Syo-Saiko-To in Japanese) (61, 62). Research has shown that its essential oil induced acute hepatotoxicity, characterized by asynchronous state, elevated heart rate, and rapid breathing (60). Furthermore, the total saponins isolated from *Radix Bupleuri* could cause significant liver damage in proportion to the dosage, resulting in hepatocyte organic lesion, impaired liver function, and hepatocyte death (60).

The variable ingredients, potency and purity of CHPs increased the risk of unintentional overdose, contamination, and adverse drug interactions, further compromising liver function (63, 64). Liver damage often develops insidiously without immediate acute symptoms, making it difficult for users to correlate problems with CHP consumption (65, 66). This challenge (67), combined with the growing popularity of herbal remedies and dietary supplements, likely leads to underreporting or misattribution of liver-related side effects (63, 66).

The liver's vital role in detoxification makes it particularly susceptible to harmful compounds from unregulated or poorly formulated CHPs (64, 68, 69). Furthermore, interactions between CHPs and conventional medications can place additional strain on the liver, especially in individuals using multiple supplements or long-term medications (70).

In summary, liver and biliary system disorders frequently appeared in CHP-related adverse event data due to certain herbs' hepatotoxic potential and the liver's vulnerability when metabolizing multiple substances. Beyond evaluating inherent herb toxicity, thorough screening for contaminants and adulterants remains crucial for product safety assessment.

“Endocrine disorders” is the fourth most commonly reported SOCs, largely contributed by the reports of Cushing's syndrome and adrenal insufficiency associated with adulterated products (see next section).

### 4.4 Adulteration of CHP

#### 4.4.1 Pain-relief products

In this study, the adulteration of undeclared drugs in CHPs seemed to be one of the main causes contributing to the adverse events (7.7%). The majority (60%) of adulterated products were marketed for treating rheumatic joint pain, knee pain, back pain, gout pain, and inflammation. These products primarily contained non-steroidal anti-inflammatory drugs (NSAIDs) such as piroxicam, ibuprofen, and diclofenac, as well as steroids including dexamethasone and prednisolone. Such adulteration presented considerable health risks, as prolonged usage of NSAIDs may lead to cardiovascular complications, including myocardial infarction and stroke (71). Furthermore, long-term unsupervised use of oral steroids can elevate blood sugar levels, potentially causing diabetes, hypertension, increased infection risk, and Cushing's Syndrome, characterized by round face, weight gain, and skin thinning (72, 73). Discontinuation of steroids without medical supervision can trigger adrenal insufficiency, a serious condition where the body's steroid hormone production becomes inadequate, resulting in fatigue, generalized weakness, musculoskeletal pain, hypotension, and potentially fits or shock (74).

There were seven case reports of serious adverse events caused by CPMs and complementary medicines adulterated with undeclared pharmaceutical drugs (25–31). The cases involved patients aged 30s−60s who developed conditions ranging from cough and abnormal blood cortisol levels to steroid-induced Cushing's syndrome. The adulterated products contained various combinations of corticosteroids (dexamethasone, prednisolone), antihistamines (chlorpheniramine, promethazine), NSAIDs (piroxicam, diclofenac, and ibuprofen), antibiotics (chloramphenicol, tetracycline, and amoxicillin), and other drugs including sildenafil and lovastatin.

These findings carry important regulatory and patient safety implications. From a regulatory standpoint, they reinforce the need for robust post-marketing surveillance, including targeted laboratory testing of CHPs, particularly those marketed for pain relief and anti-inflammatory purposes. From a patient safety perspective, healthcare professionals should maintain a high index of suspicion for undeclared drug adulteration when patients present with unexpected clinical findings after consuming CHPs and should routinely enquire about CHP use during clinical assessments. Public education on the risks of unregulated CHPs remains equally important in safeguarding public health.

#### 4.4.2 Slimming products

As the global population increasingly grapples with overweight and obesity issues, a substantial market for slimming products has emerged. These products appeal to consumers through their health-related claims, perceived safety, widespread availability, competitive pricing, and extensive marketing strategies. In this study, products marketed for weight loss were frequently found to be adulterated with sibutramine. Sibutramine, previously available as a prescription-only weight loss medication, was banned in 2010 across Singapore, the United States, the United Kingdom, and the European Union due to significant cardiovascular risks (75, 76). Its adverse effects range from elevated blood pressure and heart rate to anxiety, mood swings, dry mouth, insomnia, and constipation, with serious cases risking heart attack and stroke (77). Despite this ban, sibutramine continued to be illicitly incorporated into slimming products marketed as safe and effective (78, 79). The proliferation of social media and e-commerce platforms has facilitated easy online access to these products, potentially exposing unwitting consumers to harmful side effects. A study analyzing reports on adverse drug reactions related to herbal medicinal products and herbal supplements in the Netherlands revealed similar findings, with sibutramine adulteration in herbal slimming supplements accounting for the highest number of reports (80).

The two serious cases of cardiovascular complications from adulterated slimming products illustrated the potentially severe consequences of sibutramine adulteration (32, 33). The claims of complementary medicine “Clinic K” containing only natural ingredients while harboring undeclared sibutramine at twice the previously permitted maximum dose may give false reassurance to consumers, thinking that the product is safe for consumption. Enhanced surveillance of slimming products may be needed due to the high potential risks of adulteration and the severity of associated health risks.

### 4.5 Concomitant drug administration

In this study, only 7.8% of patients reported using both CHPs and conventional drugs concurrently. This low percentage may stem from under-reporting, as patients or healthcare professionals might either fail to recognize the significance of such information or feel hesitant to disclose it. The concurrent use of CHPs and conventional medicines could result in herb-drug interactions, potentially leading to serious adverse effects (81). Those at elevated risk of harmful herb-drug interactions include children, elderly individuals, pregnant women, and patients with chronic illnesses or compromised organ function. When healthcare professionals are unaware of concurrent CHPs use in their patients, adverse events arising from drug-herb interactions may go undetected. Hence, transparent communication between patients and their healthcare professionals about the use of both CHPs and pharmaceutical products is essential for minimizing adverse events.

### 4.6 Regulatory actions

As part of the post-market product quality surveillance programme to monitor the safety of CHPs, HSA regularly conducts risk-based sampling and laboratory testing of CHPs available in the Singapore market under its post-market product quality surveillance programme. The authority requires that CHPs are free from undeclared drugs and do not exceed the permissible legal limits for toxic heavy metals, thereby ensuring compliance with safety and quality standards. When products are found to contain undeclared drugs or excessive levels of heavy metals, HSA will take prompt regulatory actions, including product recalls, press release issuance, and prosecution of errant suppliers. The authority also maintains close collaboration with international regulatory partners to exchange critical information on adulterated products and emerging safety concerns.

### 4.7 Limitations

This study, based on retrospective analysis of spontaneous adverse event reports, has several limitations. Reports commonly lacked crucial information, such as information about the product (i.e., route of administration, indications, and therapy dates and doses), details about the adverse event (e.g., time-to-onset, relevant laboratory tests) or confounders (e.g., patient's pre-existing conditions, concomitant drugs). Many reports also omitted detailed information about herbal ingredients or product samples that were not available for testing. Without comprehensive toxicity analysis of suspected products, it was not possible to verify the presence of harmful ingredients or adulterants, nor determine how patients' underlying medical conditions might have contributed to adverse effects.

Establishing causality between the CHPs and the adverse event could be challenging, especially when confounded by incomplete case details. Hence, causality assessment often took into account the reporters' evaluation. If the product sample is available, it may be sent for laboratory analysis to screen for adulterants or toxic heavy metals, and the results could aid in causality assessment as well. Healthcare professionals documented these events as part of their professional duties when patients sought treatment.

Under-reporting by healthcare professionals was common, particularly when adverse effects resolved naturally after discontinuation, when associations between products and adverse effects were not immediately apparent, or when healthcare professionals and patients are unaware that herbal adverse events should be reported or are unsure of the reporting requirements (5, 82). Patients may also contribute to under-reporting, as individuals often hesitated to disclose their use of CHPs to healthcare professionals, primarily because they did not view these products as medicines, when products were sensitive in nature (such as slimming and sexual enhancement products) or when products were purchased over the counter without medical consultation (5, 82). Most patients obtained information about herbs and supplements from sources other than their healthcare professionals (83). Patients may incorrectly assume that such natural products were inherently safe and hence substitute such CHPs for conventional medications without informing their healthcare professionals.

As the study utilizes spontaneous adverse event reports and in the absence of product exposure data, the true incidence of adverse events with CHPs is not known.

## 5 Conclusion

This study examined 727 adverse event reports associated with CHPs between 2017 and 2023, accounting for 0.4% of all reported adverse events. Health supplements accounted for the highest number of reports (68% of total), showing only a moderate decrease from the previous 7-year period. “Skin and appendages disorders” were the most commonly reported system organ class, with glucosamine-containing products accounting for the highest number of adverse events. Products found to be adulterated (i.e., containing illegally added substances that were not declared on their labels) were most frequently indicated for pain relief. While CHPs are generally safe, this analysis underscores the concern that adulterated products, especially those from dubious sources, pose real health risks to consumers. Users should carefully monitor their response to these CHPs, especially when initiating treatment or increasing dosages. Healthcare professionals and the public should remain vigilant about potential adulteration of CHPs and be diligent in identifying adverse events. Even when causality remains unclear, healthcare professionals are strongly encouraged to report adverse events whenever they suspect an association between the CHP and the adverse event. This would enable prompt regulatory intervention when concerning patterns emerge. The continued spontaneous reporting of adverse events remains essential for monitoring the safety of CHPs and understanding associated risks. Effective safety surveillance of CHPs requires ongoing collaboration between healthcare professionals, regulators, manufacturers, and the public.

## Data Availability

The data analyzed in this study is subject to the following licenses/restrictions: Source data cannot be shared as it contains protected health information.

## References

[1] Lim MK Sadarangani P Chan HL Heng JY. Complementary and alternative medicine use in multiracial Singapore. Complement Ther Med. (2005) 13:16–24. doi: 10.1016/j.ctim.2004.11.002 15907674

[2] Lee TL. Complementary and alternative medicine, and traditional Chinese medicine: time for critical engagement. Ann Acad Med Singap. (2006) 35:749–52. doi: 10.47102/annals-acadmedsg.V35N11p749 17160186

[3] Lee GBW Charn TC Chew ZH Ng TP. Complementary and alternative medicine use in patients with chronic diseases in primary care is associated with perceived quality of care and cultural beliefs. Fam Pract. (2004) 21:654–60. doi: 10.1093/fampra/cmh613 15531625

[4] Nortier JL Vanherweghem JL. For patients taking herbal therapy—lessons from aristolochic acid nephropathy. Nephrol Dial Transplant. (2007) 22:1512–7. doi: 10.1093/ndt/gfm167 17405785

[5] Shaw D Ladds G Duez P Williamson E Kelvin C. Pharmacovigilance of herbal medicine. J Ethnopharmacol. (2012) 140:513–8. doi: 10.1016/j.jep.2012.01.051 22342381

[6] World Health Organization. WHO Guidelines on Safety Monitoring of Herbal Medicines in Pharmacovigilance Systems. (2004). Available online at: https://www.who.int/publications/i/item/9241592214 [Accessed April 21, 2025].

[7] Health Sciences Authority. Health Supplements Guidelines. (2024). Available online at: https://www.hsa.gov.sg/docs/default-source/hprg-tmhs/hs_guidelines.pdf?sfvrsn=72e4bbd0_17 [Accessed April 21, 2025].

[8] Health Sciences Authority. Guidelines on Chinese Proprietary Medicines Product Listing. (2025). Available online at: https://www.hsa.gov.sg/docs/default-source/hprg-tmhs/chinese-proprietary-medicines/pl_fsc_guidelines.pdf?sfvrsn=8da888fd_15 [Accessed April 21, 2025].

[9] Health Sciences Authority. Traditional Medicines Guidelines. (2024). Available online at: https://www.hsa.gov.sg/docs/default-source/hprg-tmhs/tm_guidelines.pdf?sfvrsn=de2ca89e_2 [Accessed April 21, 2025].

[10] Yee SK Chu SS Xu YM Choo PL. Regulatory control of Chinese Proprietary Medicines in Singapore. Health Policy. (2005) 71:133–49. doi: 10.1016/j.healthpol.2003.09.013 15607377

[11] Woolf AD. Safety evaluation and adverse events monitoring by poison control centers: a framework for herbs and dietary supplements. Clin Toxicol. (2006) 44:617–22. doi: 10.1080/15563650600795578 16905504

[12] van Breemen RB Fong HHS Farnsworth NR. Ensuring the safety of botanical dietary supplements. Am J Clin Nutr. (2008) 87:509S−13S. doi: 10.1093/ajcn/87.2.509S 18258648

[13] Jordan SA Cunningham DG Marles RJ. Assessment of herbal medicinal products: challenges, and opportunities to increase the knowledge base for safety assessment. Toxicol Appl Pharmacol. (2010) 243:198–216. doi: 10.1016/j.taap.2009.12.005 20018204

[14] Sooi LK Keng SL. Herbal medicines: Malaysian women's knowledge and practice. Evid Based Complement Alternat Med. (2013) 2013:438139. doi: 10.1155/2013/438139 24093047 PMC3777224

[15] Patel DN Low WL Tan LL Tan MMB Zhang Q Low MY . Adverse events associated with the use of complementary medicine and health supplements: an analysis of reports in the Singapore Pharmacovigilance database from 1998 to 2009. Clin Toxicol. (2012) 50:481–9. doi: 10.3109/15563650.2012.700402 22738039

[16] Xu Y Patel DN Ng SLP Tan SH Toh D Poh J . Retrospective study of reported adverse events due to complementary health products in Singapore from 2010 to 2016. Front Med. (2018) 5:167. doi: 10.3389/fmed.2018.00167 29946545 PMC6006675

[17] Health Sciences Authority. Adverse Event Reporting Form. (2022). Available online at:https://www.hsa.gov.sg/docs/default-source/hprg-vcb/adverse-events/adr_form_therapeutic-products_feb2022.pdf?sfvrsn=d048a39c_2 (Accessed May 02, 2025).

[18] World Health Organisation. The Use of the WHO-UMC System for Standardised Case Causality Assessment. (2013). Available online at: https://www.who.int/docs/default-source/medicines/pharmacovigilance/whocausality-assessment.pdf (Accessed May 02, 2025).

[19] World Health Organization. Uppsala Monitoring Centre—Accessing Global Data with VigiBase Analysis and Search Services. (2024). Available online at: https://who-umc.org/vigibase-data-access/ (Accessed May 02, 2025).

[20] WHO-Adverse Reaction Terminology (WHO-ART). Dictionary of Pharmaceutical Medicine. Vienna: Springer (2009). pp. 192–3. doi: 10.1007/978-3-211-89836-9_1467

[21] Liu SY Woo SO Koh HL. HPLC and GC-MS screening of Chinese proprietary medicine for undeclared therapeutic substances. J Pharm Biomed Anal. (2001) 24:983–92. doi: 10.1016/S0731-7085(00)00571-9 11248492

[22] Health Sciences Authority. HSA Alert: Four Products Found to Contain Potent Medicinal Ingredients Including Steroids and Banned Substance; Four Consumers Had Adverse Effects After Consuming Three of the Products. (2023). Available online at: https://www.hsa.gov.sg/announcements/press-release/hsa-alert-four-products-found-to-contain-potent-medicinal-ingredients-including-steroids-and-banned-substance (Accessed May 02, 2025).

[23] Yang F Dong X Yin X Wang W You L Ni J. Radix bupleuri: a review of traditional uses, botany, phytochemistry, pharmacology, and toxicology. Biomed Res Int. (2017) 2017:7597596. doi: 10.1155/2017/7597596 28593176 PMC5448051

[24] Health Science Authority. HSA Alert: Two Consumers Experienced Adverse Effects After Taking Products with Potent Undeclared Ingredients. (2022). Available online at: https://www.hsa.gov.sg/announcements/press-release/hsaalert-xpe-fs (Accessed May 05, 2025).

[25] Health Sciences Authority. HSA Alert: Consumer Developed Diabetes & Other Adverse Reactions After Taking ‘PHQ 1001 Khasiat Penawar Herba Qaseh Serata Herb' Which Contains Undeclared Potent Western Medicines. (2017). Available online at: https://www.hsa.gov.sg/announcements/press-release/hsa-alert-consumer-developed-diabetes-other-adverse-reactions-after-taking-‘phq-1001-khasiat-penawar-herba-qaseh-serata-herb'-which-contains-undeclared-potent-western-medicines (Accessed May 05, 2025).

[26] Health Sciences Authority. HSA Alert: ‘Lung Tan Tsao' and ‘Candy B+ *Coffee Extra Power' Found to Contain Potent Medicinal Ingredients; One Led to Adverse Effects in a Consumer*. (2020). Available online at: https://www.hsa.gov.sg/announcements/press-release/hsa-alert-lungtantsao-candybcoffee (Accessed May 14, 2025).

[27] Health Sciences Authority. HSA Alert: ‘D'sihat Herba Gout & Sendi' & ‘Yanwo Chongcao Yanyin Qinfei Huatan Dan' Found to Contain Potent Medicinal Ingredients; One Consumer Hospitalised, Another Had Serious Adverse Effects. (2023). Available online at: https://www.hsa.gov.sg/announcements/press-release/hsa-alert-d-sihat-herba-gout-sendi-yanwo-chongcao (Accessed May 14, 2025).

[28] Health Sciences Authority. HSA Alert: A Consumer Was Hospitalised in ICU and Another Developed Serious Adverse Reactions After Taking Health Products Bought Overseas. (2017). Available online at: https://www.hsa.gov.sg/announcements/press-release/hsa-alert-a-consumer-was-hospitalised-in-icu-and-another-developed-serious-adverse-reactions-after-taking-health-products-bought-overseas (Accessed May 05, 2025).

[29] Health Sciences Authority. HSA Alert: Five Products Detected to Contain Potent Medicinal Ingredients Including Steroids; Two Consumers Experienced Serious Adverse Effects. (2022). Available online at: https://www.hsa.gov.sg/announcements/press-release/hsa-alert-five-products-detected-to-contain-potent-medicinal-ingredients (Accessed May 15, 2025).

[30] Health Sciences Authority. HSA Alert: Three Products Found with Undeclared Potent Medicinal Ingredients; One Led to Serious Adverse Reaction in Consumer. (2019). Available online at: https://www.hsa.gov.sg/announcements/press-release/hsa-alert-three-products-found-with-undeclared-potent-medicinal-ingredients-one-led-to-serious-adverse-reaction-in-consumer (Accessed May 15, 2025).

[31] Health Sciences Authority. HSA Alert: Three Products Found to Contain Potent Medicinal Ingredients, Including Steroids and High Levels of Banned Substance Sibutramine; Two Consumers Developed Adverse Effects. (2023). Available online at: https://www.hsa.gov.sg/announcements/press-release/hsa-alert-three-products-found-to-contain-potent-medicinal-ingredients-including-steroids-and-high-levels-of-banned-substance-sibutramine (Accessed May 15, 2025).

[32] Health Sciences Authority. HSA Alert: ‘Clinic K' and ‘RO Slim Booster' Found to Contain Banned Substance, ‘Rozell Detox' Detected with Potent Laxative. (2020). Available online at: https://www.hsa.gov.sg/announcements/press-release/hsa-alert-clinick-roslimbooster-rozelldetox (Accessed May 15, 2025).

[33] Health Sciences Authority. HSA Alert: Four Products Detected with Potent Undeclared Ingredients; One Consumer Had a Life-Threatening Event and Severe Heart Failure. (2019). Available online at: https://www.hsa.gov.sg/announcements/press-release/hsa-alert-four-products-detected-with-potent-undeclared-ingredients-one-consumer-had-a-life-threatening-event-and-severe-heart-failure (Accessed May 15, 2025).

[34] Health Sciences Authority. HSA Alert: Four Products Sold Online Found to Contain Potent or Banned Ingredients Including High Levels of Arsenic and Sibutramine; Two Consumers Suffered Adverse Effects. (2023). Available online at: https://www.hsa.gov.sg/announcements/press-release/hsa-alert-four-adulterated-products (Accessed May 05, 2025).

[35] Ang JY Ooi GS. Abd.Aziz F, Tong SF. Risk-taking in consumers' online purchases of health supplements and natural products: a grounded theory approach. J of Pharm Policy and Pract. (2023) 16:134. doi: 10.1186/s40545-023-00645-x 37924079 PMC10623737

[36] Hassen G Belete G Carrera KG Iriowen RO Araya H Alemu T . Clinical implications of herbal supplements in conventional medical practice: a US perspective. Cureus. (2022) 14:e26893. doi: 10.7759/cureus.26893 35978741 PMC9375827

[37] Bruyère O Altman RD Reginster JY. Efficacy and safety of glucosamine sulfate in the management of osteoarthritis: evidence from real-life setting trials and surveys. Semin Arthritis Rheum. (2016) 45:S12–7. doi: 10.1016/j.semarthrit.2015.11.011 26806187

[38] Anderson JW Nicolosi RJ Borzelleca JF. Glucosamine effects in humans: a review of effects on glucose metabolism, side effects, safety considerations and efficacy. Food Chem Toxicol. (2005) 43:187–201. doi: 10.1016/j.fct.2004.11.006 15621331

[39] Woo CK. Bahna SL. Not all shellfish “allergy” is allergy! *Clin Transl Allergy*. (2011) 1:3. doi: 10.1186/2045-7022-1-3 22410209 PMC3294628

[40] Department of Statistics Singapore. Population Trends. (2023). Available online at: https://www.singstat.gov.sg/find-data/search-by-theme/population/population-and-population-structure/visualising-data/population-dashboard (Accessed May 09, 2025).

[41] Zazzara MB Palmer K Vetrano DL Carfi A Onder G. Adverse drug reactions in older adults: a narrative review of the literature. Eur Geriatr Med. (2021) 12:463–73. doi: 10.1007/s41999-021-00481-9 33738772 PMC8149349

[42] Gutierrez-Valencia M Izquierdo M Cesari M Casas-Herrero A Inzitari M Martinez-Velilla N. The relationship between frailty and polypharmacy in older people: a systematic review. Br J Clin Pharmacol. (2018) 84:1432–44. doi: 10.1111/bcp.13590 29575094 PMC6005607

[43] Rafati S Baniasadi T Dastyar N Zoghi G Ahmadidarrehsima S Salari N . Prevalence of self-medication among the elderly: a systematic review and meta-analysis. J Edu Health Promot. (2023) 12:67. doi: 10.4103/jehp.jehp_630_22 37113410 PMC10127510

[44] Chen XW Sneed KB Pan SY Cao C Kanwar JR Chew H . Herb-drug interactions and mechanistic and clinical considerations. Curr Drug Metab. (2012) 13:640–51. doi: 10.2174/1389200211209050640 22292789

[45] Choi Y and Shin HK. Adverse events associated with herbal medicine products reported in the Korea adverse event reporting system from 2012 to 2021. Front Pharmacol. (2024) 15:1378208. doi: 10.3389/fphar.2024.1378208 39498343 PMC11532164

[46] Chang HH Chiang SY Chen PC Tsai CH Yang RC Tsai CL . A system for reporting and evaluating adverse drug reactions of herbal medicine in Taiwan from 1998 to 2016. Sci Rep. (2021) 11:21476. doi: 10.1038/s41598-021-00704-w 34728662 PMC8564513

[47] Watson S Caster O Rochon PA den Ruijter H. Reported adverse drug reactions in women and men: aggregated evidence from globally collected individual case reports during half a century. eClinicalMedicine. (2019) 17:100188. doi: 10.1016/j.eclinm.2019.10.001 31891132 PMC6933269

[48] Zopf Y Rabe C Neubert A Gaßmann KG Rascher W Hahn EG . Women encounter ADRs more often than do men. Eur J Clin Pharmacol. (2008) 64:999–1004. doi: 10.1007/s00228-008-0494-6 18604529

[49] Kristoffersen AE Stub T Salamonsen A Musial F Hamberg K. Gender differences in prevalence and associations for use of CAM in a large population study. BMC Complement Altern Med. (2014) 14:463. doi: 10.1186/1472-6882-14-463 25465676 PMC4265502

[50] Alomar MJ. Factors affecting the development of adverse drug reactions. Saudi Pharm J. (2014) 22:83–94. doi: 10.1016/j.jsps.2013.02.003 24648818 PMC3950535

[51] Bishop F Lewith G. Who uses CAM? A narrative review of demographic characteristics and health factors associated with CAM use. Evid Based Complement Alternat Med. (2010) 7:11–28. doi: 10.1093/ecam/nen023 18955327 PMC2816378

[52] Barvaliya MJ Chetan AC Chandan N Ray SK Hegde HV Unger BS . Suspected cutaneous adverse drug reactions reported with traditional medicines: analysis of data for United Nations Asia region from WHO VigiBase. Front Pharmacol. (2023) 14:1088841. doi: 10.3389/fphar.2023.1088841 37324461 PMC10261983

[53] Ernst E. Adverse effects of herbal drugs in dermatology. Br J Dermatol. (2000) 143:923–9. doi: 10.1046/j.1365-2133.2000.03822.x 11069498

[54] Posadzki P Watson LK Ernst E. Adverse effects of herbal medicines: an overview of systematic reviews. Clin Med. (2013) 13:7–12. doi: 10.7861/clinmedicine.13-1-7 23472485 PMC5873713

[55] Cork MJ Danby SG Vasilopoulos Y Hadgraft J Lane ME Moustafa M . Epidermal barrier dysfunction in atopic dermatitis. J Invest Dermatol. (2009) 129:1892–908. doi: 10.1038/jid.2009.133 19494826

[56] Schnuch A Westphal G Mossner R Uter W Reich K. Genetic factors in contact allergy - review and future goals. Contact Dermatitis. (2011) 64:2–23. doi: 10.1111/j.1600-0536.2010.01800.x 21166814

[57] Health Sciences Authority. HSA ADR News Bulletin Dec 2022, Vol. 24. (2022). p. 57. Available online at: https://www.hsa.gov.sg/announcements/adr-news-bulletin-2022-december–volume-24-number-3-/ (Accessed November 27, 2025).

[58] Frenzel C Teschke R. Herbal hepatotoxicity: clinical characteristics and listing compilation. Int J Mol Sci. (2016) 17:588. doi: 10.3390/ijms17050588 27128912 PMC4881436

[59] Tarantino G Pezzullo MG. Dario di Minno MN, Milone F, Pezzullo LS, Milone M, et al. Drug-induced liver injury due to “natural products” used for weight loss: a case report. World J Gastroenterol. (2009) 15:2414–7. doi: 10.3748/wjg.15.2414 19452589 PMC2684613

[60] Liu Yamin LiZ Liu X Pan R. Review on the toxic effects of radix bupleuri. Curr Opin Complement Alternat Med. (2014) 1:3–7. Available online at: https://www.researchgate.net/publication/285309506_Review_on_the_Toxic_Effects_of_Radix_Bupleuri

[61] Itoh S Marutani K Nishijima T Matsuo S Itabashi M. Liver injuries induced by herbal medicine, Syo-saiko-to (xiao-chai-hu-tang). Digest Dis Sci. (1995) 40:1845–8. doi: 10.1007/BF02212712 7648990

[62] Hsu LM Huang YS Tsay SH Chang FY Lee SD. Acute hepatitis induced by Chinese hepatoprotective herb, xiao-chai-hu-tang. J Chin Med Assoc. (2006) 69:86–8. doi: 10.1016/S1726-4901(09)70119-4 16570576

[63] Navarro VJ Khan I Bjornsson E Seeff LB Serrano J Hoofnagle JH. Liver injury from herbal and dietary supplements. Hepatology. (2017) 65:363–73. doi: 10.1002/hep.28813 27677775 PMC5502701

[64] Stickel F Shouval D. Hepatotoxicity of herbal and dietary supplements: an update. Arch Toxicol. (2015) 89:851–65. doi: 10.1007/s00204-015-1471-3 25680499

[65] Teschke R Frenzel C Glass X Schulze J Eickhoff A. Herbal hepatotoxicity: a critical review. Br J Clin Pharmacol. (2013) 75:630–6. doi: 10.1111/j.1365-2125.2012.04395.x 22831551 PMC3575930

[66] García-Cortes M Robles-Díaz M Ortega-Alonso A Medina-Caliz I Andrade RJ. Hepatotoxicity by dietary supplements: A tabular listing and clinical characteristics. Int J Mol Sci. (2016) 17:537. doi: 10.3390/ijms17040537 27070596 PMC4848993

[67] Chalasani NP Maddur H Russo MW Wong RJ Reddy KR ACG. Clinical guideline: Diagnosis and management of idiosyncratic drug-induced liver injury. Am J Gastroenterol. (2021) 116:878–98. doi: 10.14309/ajg.0000000000001259 33929376

[68] Hussaini SH Farrington EA. Idiosyncratic drug-induced liver injury: an update on the 2007 overview. Expert Opin Drug Saf. (2014) 13:67–81. doi: 10.1517/14740338.2013.828032 24073714

[69] Posadzki P Watson L Ernst E. Contamination and adulteration of herbal medicinal products (HMPs): an overview of systematic reviews. Eur J Clin Pharmacol. (2013) 69:295–307. doi: 10.1007/s00228-012-1353-z 22843016

[70] Lam CS Hua R Au-Doung PLW Wu YK Koon HK Zhou KR . Association between potential supplement-drug interactions and liver diseases in patients with cancer: a large prospective cohort study. Clin Nutrit ESPEN. (2023) 58:152–9. doi: 10.1016/j.clnesp.2023.09.919 38057000

[71] Varga Z Sabzwari SRA Vargova V. Cardiovascular risk of nonsteroidal anti-inflammatory drugs: an under-recognized public health issue. Cureus. (2017) 9:e1144. doi: 10.7759/cureus.1144 28491485 PMC5422108

[72] Pawlosky N. Cardiovascular risk: are all NSAIDs alike? Can Pharm J. (2013) 146:80–3. doi: 10.1177/1715163513481569 23795181 PMC3676195

[73] Ericson-Neilsen W Kaye AD. Steroids: pharmacology, complications, and practice delivery issues. Ochsner J. (2014) 14:203–7. 24940130 PMC4052587

[74] Pazderska A Pearce SH. Adrenal insufficiency - recognition and management. Clin Med. (2017) 17:258–62. doi: 10.7861/clinmedicine.17-3-258 28572228 PMC6297573

[75] European Medicines Agency. European Medicines Agency Recommends Suspension of Marketing Authorisations for Sibutramine. (2010). Available online at: https://www.ema.europa.eu/en/documents/press-release/european-medicines-agency-recommends-suspension-marketing-authorisation-sibutramine_en.pdf (Accessed May 15, 2025).

[76] U.S. Food and Drug Administration. FDA Drug Safety Communication: FDA Recommends Against the Continued Use of Meridia (Sibutramine). (2010). Available online at: https://www.fda.gov/drugs/drug-safety-and-availability/fda-drug-safety-communication-fda-recommends-against-continued-use-meridia-sibutramine#Safety_Announcement (Accessed May 15, 2025).

[77] Chao AM Wadden TA Berkowitz RI Quigley K Silvestry F. The risk of cardiovascular complications with current obesity drugs. Expert Opin Drug Saf. (2020) 19:1095–104. doi: 10.1080/14740338.2020.1806234 32750250 PMC7554173

[78] Stypulkowska K Blazewicz A Maurin J Sarna K Fijalek Z. X-ray powder diffractometry and liquid chromatography studies of sibutramine and its analogues content in herbal dietary supplements. J Pharm Biomed Anal. (2011) 56:969–75. doi: 10.1016/j.jpba.2011.08.028 21899974

[79] Mathon C Ankli A Reich E Bieri S Christen P. Screening and determination of sibutramine in adulterated herbal slimming supplements by HPTLC-UV densitometry. Food Addit Contam: Part A. (2014) 31:15–20. doi: 10.1080/19440049.2013.861934 24215519

[80] van Hunsel FPAM van der Kooi D van de Koppel S Kroes BH Woerdenbag HJ. Analysis of reports on adverse drug reactions related to herbal medicinal products and herbal supplements in the Netherlands received by the National Pharmacovigilance Centre Lareb. Drug Saf. (2022) 45:651–61. doi: 10.1007/s40264-022-01180-5 35608783

[81] Hu Z Yang X Ho PCL Chan SY Heng PWS Chan E et al. Herb-drug interactions: a literature review. Drugs. (2005) 65:1239–82. doi: 10.2165/00003495-200565090-00005 15916450

[82] Barnes J. Pharmacovigilance of herbal medicines: a UK perspective. Drug Saf. (2003) 26:829–51. doi: 10.2165/00002018-200326120-00001 12959628

[83] Basaran N Pasli D Basaran AA. Unpredictable adverse effects of herbal products. Food Chem Toxicol. (2022) 159:112762. doi: 10.1016/j.fct.2021.112762 34896186

